# THE ROLE OF CARE PARTNERS IN SUPPORTING INNER STRENGTH OF PERSONS NEWLY DIAGNOSED WITH MILD COGNITIVE IMPAIRMENT

**DOI:** 10.1093/geroni/igac059.1126

**Published:** 2022-12-20

**Authors:** Brianna Morgan, Lauren Massimo, Sharon Ravitch, Jason Karlawish, Nancy Hodgson

**Affiliations:** University of Pennsylvania, Philadelphia, Pennsylvania, United States; University of Pennsylvania, Philadelphia, Pennsylvania, United States; University of Pennsylvania, Philadelphia, Pennsylvania, United States; University of Pennsylvania Perelman School of Medicine, Philadelphia, Pennsylvania, United States; University of Pennsylvania, Philadelphia, Pennsylvania, United States

## Abstract

Inner strength is one’s internal process of facing challenging circumstances, like receiving a mild cognitive impairment (MCI) diagnosis. However, the role of care partners in this process is unknown. This study explores the role of care partners in supporting inner strength at the time of diagnosis using qualitative methodologies. We interviewed persons diagnosed with MCI (N=5) at a Memory Center within 12 months and their care partners (N=5). We analyzed data in NVivo using reflexive thematic analysis methods. Trustworthiness was maintained through vetted semi-structured interview guides, verbatim transcription, field notes, peer analysis, and audit trails. Care partners supplant cognitive losses (e.g., redistribution of self, organizing and coordinating) to allow the person with MCI to thrive, which is built on a foundation of the care partner’s personal resources and the nature of their relationship with the person with MCI. Implications include incorporating care partners into diagnostic processes and tailoring caregiving supports.

